# The detrimental impacts of smart technology device overuse among school students in Kuwait: a cross-sectional survey

**DOI:** 10.1186/s12887-020-02417-x

**Published:** 2020-11-16

**Authors:** Ali Jasem Buabbas, Madawi Anwar Al-Mass, Basma Awad Al-Tawari, Mohammad Abbas Buabbas

**Affiliations:** 1 Department of Community Medicine and Behavioural Sciences, Faculty of Medicine, Kuwait University, P.O. Box 24923, 13110 Safat, Kuwait; 2 Ministry of Health, Pediatrics Board, Kuwait Institute for Medical Specialization, Kuwait City, Kuwait; 3 Department of Physical Education, College of Basic Education, The Public Authority for Applied Education and Training, Kuwait City, Kuwait

**Keywords:** Detrimental impact, Smart technology devices, Overuse, Health, School-aged children

## Abstract

**Background:**

Children and adolescents are becoming the most prolific users of smart technology (ST) devices due to the numerous advantages presented by these devices. However, the overuse of ST devices can have detrimental impacts on health. Therefore, the aim of this study was to investigate the pattern of ST device use among school students in Kuwait and the possible associated health problems.

**Methods:**

This cross-sectional survey used a pretested questionnaire to collect data from students of different educational levels within the governmental sector: primary, secondary and high school. Chi-square tests were applied to find associations or significant differences between the categorical variables, in which *p* < 0.05 was considered statistically significant.

**Results:**

This study included 3015 students, of whom 53.6% were female. The sample had an equal distribution of primary (33.8%), secondary (32.4%) and high school students (33.8%). Almost all of the participants (99.7%) owned a ST device, chiefly smartphones (87.7%). Most of the students used ST devices for > 4 total hours per day on average, which is categorised as “overuse”. Among those overusing ST devices, the symptoms most commonly experienced included headaches (35.0%), sleep disturbances (36.6%) and neck/shoulder pain (37.7%). Students who used ST devices for < 1 h per session experienced eye-related problems. Moreover, students who played sports on a regular basis were more likely to spend less time per session on ST devices. The prolonged use of ST devices was associated with higher reporting of seizures, eye squints and transient vision loss.

**Conclusion:**

The overuse of ST devices per day and per session by school-aged children has the potential to have a detrimental impact on their health, as has been noticed among students in Kuwait. Healthcare professionals, school authorities and parents could use these results to plan strategies to change ST device use behaviours among schoolchildren.

**Supplementary Information:**

The online version contains supplementary material available at 10.1186/s12887-020-02417-x.

## Background

Smart technology (ST) devices, including smartphones and tablets, are electronic gadgets able to offer various features that users need in their daily activities, including phone calls, built-in digital cameras, Internet access, telecommunication media and software applications designed for multiple purposes. ST devices have similar capabilities to a personal computer or laptop, providing users with access to the internet through their mobile phones, wherein most of the online activities are based on online communication (e.g. WhatsApp), social media (e.g. Instagram, Twitter and Snapchat) and entertainment (e.g. games, films and music). The availability of these functions encourages people to use these devices at any time and from anywhere, and for long periods of time to the extent that they become dependent on them. Overuse or addiction is referring to spend too much time on smart devices until it negatively affects the user’s daily life [1]. The Canadian Pediatrics Society (CPS) has categorized ST devices use for > 4 h per day as overuse [2]. The addiction on internet use is a major reason of adopting people for smart devices [1]. Several studies have reported that mental health is influenced by patterns of internet use [3, 4] as well as the physical health [1]. It is reported that the most frequent internet users are adolescents and young adults and they may use it without awareness of its potential negative consequences [5]. Hence, they are more vulnerable to the adverse effects of SP overuse [5, 6]. Pew Research Center reported that nearly 95% of teens in the United States have access to smartphones, and many of them have concerns about overusing them [7]. A study in South Korea found that smartphone use was more common among the age group of 10–20 years old than the age group of 20–30 years old [8]. Another study reported that adolescent and elementary school students have addictions to the use of smartphones similar to those seen among adults [9]. This is due to that children and teenagers have less-developed self-control compared to adults [9, 10]. Regardless of the advantages of ST devices, detrimental effects are becoming apparent in society.

### Overuse of ST devices and health-related problems

Several studies have been conducted on the overuse of ST devices and its impact on individuals’ health, both physical and psychological [11, 12]. The health problems found to be associated with ST device use include neck/shoulder and lower-back pain [10], headaches [13, 14], visual problems [1, 8], and obesity [15, 16]. Using ST devices restricts individuals’ movement, particularly for children and youths. This is considered a factor that is contributing to the increasing obesity rate among children, which is considered an epidemic [16]. A systematic review found that sleep disturbances (duration and timing) among school-aged children and adolescents was positively associated with screen time [17]. In addition, age, gender, type of screen exposure and day of the week were associated with sleep disturbances.

Various health problems can arise due to ST device overuse, which are associated with several predictive factors. Research have shown that excessive smartphone use among young adults has associated with health-related problems, such studies as in India, Karachi, and Sweden, wherein its commonly found that vision, hearing, and concentration, and reduced of physical activity are negatively affected [18].

Currently, the ST devices use are spreading out not only among the young generation, but also among the childhood and without constraints, in which the impact of overuse could be expected to be very detrimental on their health. To date, neither research done in this topic among school students, nor local guidelines for ST use in Kuwait or Gulf countries are available. Therefore, this study aimed to investigate the prevalence of and the factors associated with ST device use among school students in the state of Kuwait and to assess the health-related side effects. The outcomes of this study will display the pattern of ST use and associated side effects of use in students to help develop recommendations that fit local culture and lifestyle. These recommendations should be communicated to all educators and health care practitioners to guide family implementation to minimize likely detrimental impacts of ST.

## Methods

### Study design, sampling technique and population

This cross-sectional survey enrolled school students aged 6–18 years old attending Kuwait Ministry of Education primary, secondary and high schools. The study covered all six educational regions in Kuwait: Asimah (Capital), Farwaniyah, Hawally, Jahra, Ahmadi and Mubarak Al-Kabeer. The total number of governmental schools is 593, excluding nursery schools and special needs schools. These are categorised based on educational level and gender. A two-stage random cluster sampling approach was used. In total, 72 schools were selected. In each of the six educational regions, two primary schools, two secondary schools and two high schools for each gender were randomly selected. In each school, two classes were selected randomly, with an average of 23 students in each class. In total, 144 classes were selected.

### Research instrument and data collection

A questionnaire survey based on an extensive review of related research articles was developed in order to collect data. Databases used were Google Scholar and PubMed. ST devices use guidelines by CPS were also part of the review. The keywords used in the search were “smartphone”, “health effects”, “students”, “headache”, “pain”, “sleep disturbances”, “physical activity” and “obesity”. The questions were phrased and reviewed by a research team consisting of one assistant professor from the Department of Community Medicine and Behavioural Sciences at Kuwait University’s Faculty of Medicine, one consultant paediatrician with two residents on the Pediatrics Board from the Kuwait Ministry of Health. Thereafter, a pilot study was performed with 15 students: five from each school level solely to test the content validity and to ensure the suitability and readability of the questions, and minor modifications were made during this process. The questionnaire was translated into Arabic because it’s the official language in Kuwait, and translation was performed by the translation office in the faculty of medicine at the Kuwait University. The data collection was professionally performed by two academic researchers, one of whom was the principle investigator. Both of them had skills in conducting this process and knowledge of the research themes. The questionnaire was distributed by hand to the students, in which the researchers waited to collect the completed questionnaires.

The questionnaire consisted of four sections (Additional file 1). Section 1 included questions about the student’s socio-demographic data, educational level, last grade point average (GPA), parents’ educational levels, total family income per month and educational region. Section 2 included questions about the student’s height, weight, physical activity rating and level of sport practised on a regular basis. Section 3 included questions about ST device use, such as the types of ST devices owned, the average total hours of ST device use per day, the average screen time spent on ST devices per session, the most common time during the day for ST device use and the purpose of using ST devices. Section 4 focused on health problems, divided into two sub-sections: (a) questions about any previously diagnosed (by a professional) health problems, such as epilepsy, headaches, migraines, visual impairments and back injuries; and (b) questions about whether the student had ever experienced any of the following health problems before or after adopting ST devices: convulsions, near-sightedness, strabismus, dry eyes, blurry vision, transient vision loss, headaches, loss of concentration, sleep disturbances, neck/shoulder pain and lower-back pain.

The questions were designed based on the most common impacts of ST use found in the literature. These impacts were related to students’ health and education. Questions about patterns of ST use (section 3) were developed based on international guidelines and cut-off points. The divisions of ST use were adapted from the CPS statement, where moderate use was defined as 2–4 h/day [2]. Therefore, in this study ST overuse was defined as use more than 4 h/day. The question about ST use per session was formulated based on the Canadian Association of Optometrists recommendation to use ST for 30–60 min per session [19].

### Ethical considerations

Ethical approval was granted by the Research Ethics Committee at the Kuwait Ministry of Health (reference number: 885). Written informed consent was obtained from a parent for the participants included in the study.

### Statistical analysis

The data management, analysis and graphical presentation were carried out using the computer software Statistical Package for the Social Sciences (SPSS), version 24.0 (IBM Corp.). The descriptive statistics are presented as numbers and percentages for the categorical variables. The quantitative variables (age and scores on factors related to ST device use) are presented as means and standard deviations (SDs) with a 95% confidence interval (CI). Chi-square tests were applied to find associations or significant differences between the categorical variables, in which *p* < 0.05 was considered statistically significant.

## Results

### Students’ socio-demographic data

A total of 3015 students (out of 3168) completed and returned the questionnaire. The return rate was 95.2%. In this sample, 53.6% of the students were female (Table 1). The sample had an equal distribution of primary, secondary and high school students and of the Kuwait educational regions: Asimah, Hawally, Mubarak Al-Kabeer, Farwaniyah, Jahra and Ahmadi. The majority of the students were Kuwaiti. The final grades during the previous semester included A (38.1%), B (31.1%), C (20.0%), D (6.8%) and F (2.4%). The majority of the parents had a bachelor’s degree and reported an income of more than 1000 KD per month. The majority of the students (67.5%) were of normal weight (weight within the 5th–95th centiles, according to the US Centers for Disease Control and Prevention (CDC) growth charts), while 24.5% were obese and 7.9% were underweight. Less than half (44.1%) of the students reported playing sports on a regular basis (three times or more per week).

**Table 1:** Socio-demographic characteristics of the study sample (*n* = 3015)

Variable	*n* (%)
**Gender**	
Male	1399 (46.4)
Female	1616 (53.6)
**School**	
Primary	1019 (33.8)
Secondary	976 (32.4)
High school	1020 (33.8)
**Educational region**	
Asimah	482 (16.0)
Hawally	524 (17.4)
Mubarak Al-Kabeer	525 (17.4)
Farwaniyah	478 (15.9)
Jahra	529 (17.5)
Ahmadi	477 (15.8)
**Nationality**	
Kuwaiti	2588 (85.8)
Non-Kuwaiti Arab	427 (14.2)
**Last semester GPA**	
A	1150 (38.1)
B	937 (31.1)
C	603 (20.0)
D	205 (6.8)
F	71 (2.4)
**Father’s education**	
Secondary school or lower	232 (7.7)
High school	585 (19.4)
Diploma	365 (12.1)
Bachelor	1162 (38.5)
Postgraduate	337 (11.2)
**Mother’s education**	
Secondary school or lower	226 (7.5)
High school	524 (17.4)
Diploma	435 (14.4)
Bachelor	1338 (44.4)
Postgraduate	189 (6.3)
**Family income (KD per month)**	
Less than 500	36 (1.2)
500–1000	259 (8.6)
1000–2000	296 (9.8)
More than 2000	292 (9.7)

### Students’ ownership of ST devices and patterns of use

In the total sample, 99.7% reported owning a ST device, mostly smartphones (87.7%). In this study, ST device use was divided into three types: overuse, moderate use and less use. The pattern of use was described by the hours of use per day and the average time spent on the ST device per session.

The prevalence of ST overuse was 59.9% (Table 2). Moreover, most of the students (44.3%) spent < 1 h per session on ST devices. Students used ST devices throughout the day, with more use during the evening (61.2%) and at bedtime (58.0%).

**Table 2:** Hours of ST device use per day among school students in Kuwait and associated factors (*n* = 3015, row %)

Variable	< 2 h	2–4 h	> 4 h	***P***-value
	*n* (%)	*n* (%)	*n* (%)	
**Gender**				
Male	192 (13.9)	363 (26.2)	829 (59.9)	0.107
Female	261 (16.2)	383 (23.8)	964 (60.0)	
**School**				
Primary	255 (25.4)	246 (24.5)	504 (50.1)	< 0.001
Secondary	119 (12.2)	232 (23.8)	623 (64.0)	
High school	79 (7.8)	268 (26.5)	666 (65.7)	
**Educational region**				
Asimah	61 (12.7)	130 (27.1)	288 (60.1)	0.024
Hawally	74 (14.2)	146 (28.0)	301 (57.8)	
Mubarak Al-Kabeer	68 (13.1)	133 (25.6)	319 (61.3)	
Farwaniyah	79 (16.7)	114 (24.2)	279 (59.1)	
Jahra	78 (14.8)	110 (20.9)	338 (64.3)	
Ahmadi	93 (19.6)	113 (23.8)	268 (56.5)	
**Nationality**				
Kuwaiti	349 (13.6)	604 (23.5)	1612 62.8)	< 0.001
Non-Kuwaiti Arab	104 (24.4)	142 (33.3)	181 (42.4)	
**Last semester GPA**				
A	238 (20.8)	324 (28.3)	581 (50.8)	< 0.001
B	111 (11.9)	209 (22.5)	609 (65.6)	
C	63 (10.5)	155 (25.9)	381 (63.6)	
D	27 (13.3)	35 (17.2)	141 (69.5)	
F	9 (12.9)	17 (24.3)	44 (62.9)	
**Father’s education**				
Secondary school or lower	31 (13.4)	54 (23.4)	146 (63.2)	0.004
High school	72 (12.4)	121 (20.8)	388 (66.8)	
Diploma	48 (13.2)	91 (25.1)	224 (61.7)	
Bachelor	192 (16.7)	314 (27.3)	645 (56.0)	
Postgraduate	40 (11.9)	89 (26.6)	206 (61.5)	
**Mother’s education**				
Secondary school or lower	35 (15.6)	54 (24.1)	135 (60.3)	0.001
High school	65 (12.5)	118 (22.7)	336 (64.7)	
Diploma	42 (9.7)	106 (24.4)	287 (66.0)	
Bachelor	224 (16.9)	358 (27.0)	746 (56.2)	
Postgraduate	29 (15.4)	40 (21.3)	119 (63.3)	
**Family income (KD per month)**				
Less than 500	13 (36.1)	9 (25.0)	14 (38.8)	0.005
500–1000	51 (19.7)	67 (25.9)	141 (54.4)	
1000-2000	42 (14.2)	85 (28.7)	169 (57.1)	
More than 2000	34 (11.7)	88 (30.2)	169 (58.1)	
**Weight [M (IQR)]** ^a^	49 kg (26)	55 kg (24)	55 kg (24)	< 0.001
**Sports on a regular basis**				
Yes	226 (17.3)	365 (27.9)	718 (54.9)	< 0.001
No	222 (13.4)	376 (22.6)	1063 (64.0)	
**Seizures**				
Yes	6 (12.8)	8 (17.0)	33 (70.2)	0.33
No	447 (15.2)	738 (25.1)	1760 (59.8)	
**Transient vision loss**				
Yes	15 (11.9)	22 (17.5)	89 (70.6)	0.041
No	438 (15.3)	724 (25.3)	1704 (59.5)	
**Eye flashes**				
Yes	43 (10.8)	80 (20.1)	275 (69.1)	< 0.001
No	410 (15.8)	666 (25.7)	1518 (58.5)	
**Eye dryness**				
Yes	72 (13.4)	119 (22.1)	348 (64.6)	0.053
No	381 (15.5)	627 (25.6)	1445 (58.9)	
**Blurred vision**				
Yes	87 (14.0)	114 (18.4)	419 (67.6)	< 0.001
No	366 (15.4)	632 (26.6)	1374 (57.9)	
**Near-sightedness**				
Yes	38 (12.1)	69 (22.0)	207 (65.9)	0.065
No	415 (15.5)	677 (25.3)	1586 (59.2)	
**Eye squints**				
Yes	9 (13.4)	17 (25.4)	41 (61.2)	0.925
No	444 (15.2)	729 (24.9)	1752 (59.9)	
**Headaches**				
Yes	124 (11.8)	251 (23.9)	677 (64.4)	< 0.001
No	329 (17.0)	495 (25.5)	1116 (57.5)	
**Sleep disturbances**				
Yes	137 (12.5)	214 (19.5)	748 (68.1)	< 0.001
No	316 (16.7)	532 (28.1)	1045 (55.2)	
**Neck/shoulder pain**				
Yes	138 (12.2)	271 (23.9)	724 (63.9)	< 0.001
No	315 (16.9)	475 (25.6)	1069 (57.5)	
**Lower-back pain**				
Yes	48 (8.6)	113 (20.2)	398 (71.2)	< 0.001
**No**	405 (16.6)	633 (26.0)	1395 (57.3)	
**Loss of concentration**				
**Yes**	75 (10.3)	155 (21.3)	499 (68.4)	< 0.001
**No**	378 (16.7)	591 (26.1)	1294 (57.2)	
**Obesity**				
**Yes**	38 (9.3)	96 (23.5)	275 (67.2)	< 0.001
**No**	415 (16.1)	650 (25.2)	1518 (58.8)	

^a^ Mann–Whitney U test

*M* Median, *IQR* Interquartile range

With regard to activities done on ST devices, 45.1% of the students ranked social media applications and 30.9% ranked watching videos as their number one activity.

### Use of ST devices and health-related complaints

**Fig. 1:**
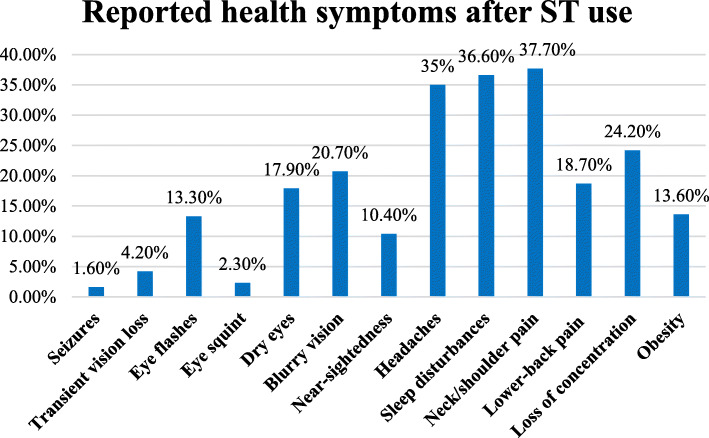
Percentages of the health symptoms reported after ST device use

The students were asked about the symptoms they had experienced after ST use and whether they were diagnosed with these symptoms before devices use. The results revealed that headaches (35.0%), sleep disturbances (36.6%) and neck/shoulder pain (37.7%) were the most commonly experienced symptoms after use (Fig. 1). Others included seizures, transient vision loss, eye flashes, eye squinting, dry eyes, blurry vision, near-sightedness, lower-back pain, loss of concentration and obesity. Before ST use, 10.1% had a pre-existing diagnosis of headaches, 0.4% had epilepsy, 17.9% had eye dryness, 3.3% had near-sightedness, 6.3% had blurred vision and 6.5% had obesity.

### Hours of use of ST devices and associated factors

The level of education was positively associated with hours of ST device use. High school students were more likely to overuse ST devices compared to younger students (*p* < 0.001; Table 2). The results show that ST device overuse was seen across all educational regions; however, it was highest in Jahra (64.3%) and lowest in Ahmadi (56.5%; *p* = 0.024). In addition, Kuwaiti students were more likely to spend a longer time on ST devices compared to non-Kuwaiti students (*p* < 0.001). Students who had achieved A grades overused ST devices less (50.8%) than students who had achieved the lower grades of B (65.6%), C (63.6%), D (69.5%) and F (62.9%) (*p* = 0.004). Furthermore, across the higher-income categories, more students used ST devices for > 4 h compared to the lowest-income group (< 500 KD per month) (*p* = 0.005), and underweight students overused ST devices more than normal and obese students did (*p* < 0.006). In addition, students who engaged in sport on a regular basis were less likely to overuse ST devices compared to those students who did not (*p* < 0.001).

The self-reported health-related signs and symptoms significantly associated (*p* < 0.001) with ST device overuse included transient vision loss, eye flashes, blurred vision, headaches, sleep disturbances, neck and shoulder pain, lower-back pain, loss of concentration and obesity (Table 2).

### Hours of ST use per session and associated factors

Across all educational levels, most of the students used ST devices for less than 1 h per session, but more primary school students (29.2%) used ST devices for prolonged periods (> 2 h at a time) compared to secondary (26.6%) and high school students (17.5%; *p* < 0.001) (Table 3). Across all educational regions, the majority of the students (40.9–49.1%) spent < 1 h on ST devices per session. However, in Ahmadi, the majority (42.8%) spent 1–2 h per session on their devices (*p* < 0.001). Students in the lowest-income group tended to spend less time on ST devices per session compared to higher-income groups (*p* < 0.001). Students who played sports on a regular basis were more likely to spend less time per session on ST devices (*p* < 0.001). The prolonged use of ST devices per session was associated with self-reporting of seizures (*p* = 0.044) and eye squints (*p* = 0.011). In addition, transient vision loss was reported more in the 1–2 h (36.5%) and > 2 h groups (33.3%) than the < 1-h group (30.2%; *p* = 0.003). In contrast, students who used ST devices for < 1 h per session experienced the following symptoms more than the other groups: eye dryness (41.4%, *p* = 0.037), blurred vision (42.2%, *p* = 0.011), near-sightedness (38.6%, *p* = 0.009), headaches (41.7% *p* = 0.004), lower-back pain (39.4%, *p* < 0.001), loss of concentration (41.2%, *p* < 0.001) and obesity (37.1%, *p* = 0.001) (Table 3).

**Table 3:** Hours of ST device use per session among school students in Kuwait and associated factors (*n* = 3015, row %)

Variable	< 1 h	1–2 h	> 2 h	***P***-value
	*n* (%)	*n* (%)	*n* (%)	
**Gender**				
Male	613 (44.5)	430 (31.2)	335 (24.3)	0.986
Female	707 (44.2)	503 (31.4)	725 (24.4)	
**School**				
Primary	426 (42.9)	278 (28.0)	290 (29.2)	< 0.001
Secondary	381 (39.2)	332 (34.2)	258 (26.6)	
High school	513 (50.6)	323 (31.9)	177 (17.5)	
**Educational region**				
Asimah	233 (48.6)	143 (29.9)	103 (21.5)	< 0.001
Hawally	256 (49.1)	153 (29.4)	112 (21.5)	
Mubarak Al-Kabeer	211 (40.8)	155 (30.0)	151 (29.2)	
Farwaniyah	211 (44.9)	147 (31.3)	112 (23.8)	
Jahra	257 (40.9)	135 (25.8)	132 (25.2)	
Ahmadi	152 (32.5)	200 (42.8)	115 (24.6)	
**Nationality**				
Kuwaiti	1092 (42.8)	812 (31.8)	649 (25.4)	< 0.001
Non-Kuwaiti Arab	228 (53.6)	121 (28.5)	76 (17.9)	
**Last semester grade**				
A	520 (45.9)	346 (30.6)	266 (23.5)	0.173
B	374 (40.4)	313 (33.8)	239 (25.8)	
C	278 (46.5)	172 (28.8)	148 (24.7)	
D	100 (49.0)	63 (30.9)	41 (20.1)	
F	31 (43.7)	23 (32.4)	17 (23.9)	
**Father’s education**				
Secondary school or lower	99 (43.0)	81 (35.2)	50 (21.7)	0.715
High school	248 (43.1)	185 (32.2)	142 (24.7)	
Diploma	152 (41.9)	121 (33.3)	90 (24.8)	
Bachelor	532 (46.3)	355 (30.9)	261 (22.7)	
Postgraduate	139 (41.6)	111 (33.2)	84 (25.1)	
**Mother’s education**				
Secondary school or lower	98 (43.8)	73 (32.6)	53 (23.7)	0.013
High school	222 (42.9)	161 (31.1)	134 (25.9)	
Diploma	158 (36.3)	169 (38.9)	108 (24.8)	
Bachelor	623 (47.3)	405 (30.7)	290 (22.0)	
Postgraduate	84 (45.2)	56 (30.1)	46 (24.7)	
**Family income (KD per month)**				
Less than 500	22 (61.1)	9 (25.0)	5 (13.9)	0.001
500–1000	111 (43.2)	85 (33.1)	61 (23.7)	
1000–2000	113 (38.2)	98 (33.1)	85 (28.7)	
More than 2000	93 (32.0)	113 (38.8)	85 (29.2)	
**Weight**				
Underweight	116 (49.6)	66 (28.2)	52 (22.2)	0.145
Normal	868 (44.2)	616 (31.4)	478 (24.4)	
Obese	311 (43.5)	218 (30.5)	186 (26.0)	
**Sports on a regular basis**				
Yes	636 (48.8)	391 (30.0)	276 (21.2)	< 0.001
No	675 (40.8)	537 (32.4)	444 (26.8)	
**Seizures**				
Yes	14 (30.4)	14 (30.4)	18 (39.1)	0.044
No	1306 (44.5)	919 (31.3)	707 (24.1)	
**Transient vision loss**				
Yes	38 (30.2)	46 (36.5)	42 (33.3)	0.003
No	1282 (45.0)	887 (31.1)	683 (23.9)	
**Eye flashes**				
Yes	182 (45.4)	113 (28.2)	106 (26.4)	0.299
No	1138 (44.2)	820 (31.8)	619 (24.0)	
**Eye dryness**				
Yes	223 (41.4)	161 (29.9)	154 (28.6)	0.037
No	1097 (45.0)	772 (31.6)	571 (23.4)	
**Blurred vision**				
Yes	261 (42.2)	179 (28.9)	179 (28.9)	0.011
No	1059 (44.9)	754 (32.0)	546 (23.1)	
**Near-sightedness**				
Yes	120 (38.6)	94 (30.2)	97 (31.2)	0.009
No	1200 (45.0)	839 (31.5)	628 (23.5)	
**Eye squints**				
Yes	20 (29.4)	22 (32.4)	26 (38.2)	0.011
No	1300 (44.7)	911 (31.3)	699 (24.0)	
**Headaches**				
Yes	437 (41.7)	318 (30.4)	292 (27.9)	0.004
No	883 (45.7)	615 (31.8)	433 (22.4)	
**Sleep disturbances**				
Yes	457 (41.7)	307 (28.0)	331 (30.2)	< 0.001
No	863 (45.8)	626 (33.2)	394 (20.9)	
**Neck and shoulder pain**				
Yes	485 (42.9)	351 (31.1)	294 (26.0)	0.229
No	835 (45.2)	582 (31.5)	431 (23.3)	
**Lower-back pain**				
Yes	220 (39.4)	164 (29.3)	175 (31.3)	< 0.001
No	1100 (45.5)	769 (31.8)	550 (22.7)	
**Loss of concentration**				
Yes	300 (41.2)	200 (27.4)	229 (31.4)	< 0.001
No	1020 (45.4)	733 (32.6)	496 (22.1)	
**Obesity**				
Yes	150 (37.1)	129 (31.9)	125 (30.9)	0.001
No	1170 (45.5)	804 (31.2)	600 (23.3)	

## Discussion

### Hours of ST device use and socio-demographic factors

The overuse of ST devices is now a worldwide phenomenon that has been linked by many studies to negative health impacts, particularly among children and adolescents. A study conducted in Lebanon reported ST device use of an average of 5 and 7 hours per day among children and adolescents, respectively [20]. This was similar to the findings of the present study, which revealed that high school students were more likely to overuse ST devices. Nevertheless, research shows that adolescents are less prone to the side effects of ST overuse [2]. Moreover, it was observed that students in the lowest-income group reported spending less time on ST devices per day and session compared to the higher-income groups. While one study reported that students from higher-income families spent more time on their mobile phones [21], another found that lower-income students used their mobile phones more often [22]. In a study conducted in Pakistan, the majority of students aged 5–16 years old (69%) reported ST device use of less than 2 h per day [23]. Perhaps the lifestyles and economic statuses of the people in these studies are the factors behind this difference in ST device use.

### Pattern of ST device use and detrimental health impacts

#### Musculoskeletal problems

With regard to the health impacts of ST device use, all the participants in the study conducted in Lebanon described having neck pain, 69% reported shoulder pain and 61% had lower-back pain [20]. The students were asked to demonstrate how they used these devices, and all of them strongly flexed their neck, which is referred to as “text neck”. A study conducted in Shanghai reported a significant increase in the prevalence of neck/shoulder and lower-back pain among high school students who used mobile phones for longer than 2 h per day [10]. Similarly, in the present study, a significant number of students who overused ST devices complained of neck and lower-back pain.

#### Eye problems

It was found that eye problems, which include transient vision loss, eye flashes, eye squinting and blurry vision were associated with ST device overuse. Further studies are required to assess the association of these symptoms with ST use. Unlike the current study, in Korea and Lebanon, in which other symptoms were associated with ST use, such as eye dryness and near-sightedness [8, 20]. This can be attributed to the fact that students’ eyes are forced to focus on close objects [20].

#### Headaches

In the present study, headaches were among the most commonly reported symptoms and were associated with ST device overuse. This was also reported in university students in Jordan and India, wherein 99.5 and 51.5% of students experienced headaches while using mobile phones, respectively [13, 24]. The association between headache and ST use is not clear. However; it was suggested that exposure to electromagnetic waves may affect the dopamine-opiate system and the integrity of the blood-brain barrier [25].

#### Lack of concentration

The findings of this study revealed a high prevalence of a lack of concentration and sleep disturbances among school students, compared to college students in India [13], in which frequent mobile interruptions from others was stated as one of the causes of a lack of concentration, which prevented students from completing their academic activities [13]. This had led to a decline in academic performance, as evidenced by the lower exam grades for some of the students [13]. In the present study, when comparing the high achievers’ use of ST devices with that of students with lower grades, it was found that half of the A-grade students (50.8%) were overusing ST devices compared to the others. However, a statement by CPS argued that if ST devices are used appropriately, screen media could improve children’s academic performance by enriching their knowledge and literacy skills. In addition, screen-based programs can encourage both autonomous and collaborative learning [2].

#### Sleep disturbances

Good night-time sleep is crucial for children’s and adolescents’ health and development, as it affects attention, behaviour, and overall mental and physical health [26]. This can be disrupted by ST device use, especially in the late hours of the day [7, 27–29]. The blue light of ST devices’ screens reduces the production of melatonin, the hormone that regulates sleep/wake cycles [13]. In the present study, sleep disturbances were reported by two-thirds of the students who overused ST devices. A study in India found that sleep disturbances, including delayed sleep onset and interrupted sleep, were reported by 35.4% of college students who used cell phones [13]. In China, a study of secondary school students found that playing on mobile phones was inversely associated with sleep duration and bedtime, as well as associated with difficulties with daytime tiredness and maintaining sleep at night [26]. A systematic review study indicated that there is a weak-to-moderate correlation between sleep and problematic to smartphones use, where further experimental studies are required to accurately study the impact of smartphones use on sleep [30].

#### Physical activity

The present study found that students who were physically active spent fewer hours on ST devices per day and less time per session. Thus, physical activity can be considered protective against the overuse of ST devices, although there is a controversy in the literature in this regard. While some studies have found that screen time is associated with a reduction in physical activity, other studies have suggested that reducing screen time does not necessarily increase the time spent on physical activity [31]. Moreover, children who are inactive tend to spend more time using ST devices, which could explain the inverse relationship between the use of ST devices and physical activity [31, 32].

#### Weight

This study found that about two-thirds of the students who overused ST devices ‘self-reported’ obesity after use. When a more objective method was used (weight was measured and plotted on CDC growth charts), it was found that underweight students were overusing ST devices more than normal weight and obese students. This might be explained by the idea that some students are so preoccupied with their phones that they omit meals and eventually develop low appetites, as reported by 20% of college students in a study conducted in India [13]. However, existing evidence suggests that ST device use usually contributes to obesity, not lower weight [15]. Screen-based media viewing encourages indiscriminate eating and greater caloric intake, as it can supress satiety cues [2, 15]. In addition, advertisements for food products, to which students might be exposed via ST device use, could increase children’s preferences for such products and eventually increase purchases of them [15]. In addition, a study in China found that addiction to smartphone device use among children could be a predictive factor for hypertension and obesity [33].

### Time of ST device use per session and health impacts

In the present study, an association was found between the presence of symptoms and the hours of ST device use per session, including higher reporting of seizures, eye squints and transient vision loss. Eye-related problems (eye dryness, blurred vision and near-sightedness), headaches, lower-back pain, loss of concentration and obesity were reported more among those who used ST devices for an hour or less at a time. This may be explained by the fact that most of the sample in this study used ST devices for less than an hour per session. The association between the length of ST device use per session and its effects on health has not been tested by other studies.

### Strengths and limitations

This study is the first of its kind in the region to investigate the detrimental impacts of ST device use among school-aged students in Kuwait governmental schools. The study had a large sample size and a high response rate (95.1%). However, the study was limited to students from the governmental education sector, so the results cannot be generalised to students at private schools or special needs schools. Furthermore, some of the questions asked the students to recall their past experiences of health-related symptoms, which could be subjected to recall bias. Moreover, those symptoms should not be attributed to ST use alone as confounding factors were not accounted for due to the cross-sectional nature of the study. Further longitudinal studies are needed to confirm the present findings. Due to the lack of research among similar populations in the region, most of the results were compared with related topics but with populations that could have different cultures, lifestyles, socioeconomic conditions and environments.

## Conclusion

This study concluded that the overuse of ST devices (in terms of both the hours of use per day and the time per session) among school-aged children could have detrimental impacts on their health, and this was noted among students in Kuwait. Healthcare professionals, school authorities and parents could use these results to plan strategies to change ST device use behaviours among schoolchildren.

Recommendations based on the findings of this study include: (1) schoolchildren should use ST devices for less than 4 h a day and reduce their screen time to no more than half an hour per session; (2) schoolchildren should avoid the use of ST devices at bedtime; (3) parents should socialise with their children to reduce their children’s screen time and to improve their patterns of ST device use; (4) schoolchildren should take regular breaks (every 30–60 min) when using ST devices and should have regular eye exams, as recommended by the Canadian Association of Optometrists [19]; (5) Health care practitioners in Kuwait should be encouraged to routinely discuss ST use guidelines with parents, emphasize on the importance of physical activity and healthy diet for children; and (6) public health solutions should be developed by responsible authorities to promote health, awareness, and safe use of ST devices among children, considering the CPS guidelines.

## Supplementary Information

**Additional file 1.** A questionnaire-English version.

## Data Availability

The datasets used and/or analysed during the current study are available from the corresponding author on reasonable request.

## Abbreviations

CDC: Centers for Disease Control and Prevention
CI: Confidence interval
CPS: Canadian Pediatrics Society
SD: Standard deviation
SPSS: Statistical Package for the Social Sciences
ST: Smart technology
GPA: Grade point average
AAP: American Academy of Pediatrics
