# Supplementary material for: The detrimental impacts of smart technology device overuse among school students in Kuwait: a cross-sectional survey
Source: BMC Pediatr. 2020 Nov 16;20:524. doi: 10.1186/s12887-020-02417-x (PMC7667779; doi:10.1186/s12887-020-02417-x)
Supplement: Supplementary file 1 — Additional file 1. A questionnaire-English version. [file 12887_2020_2417_MOESM1_ESM.docx]

**Section 1: Socio-demographic data**

**1. Gender:**

⬜_1_ Male ⬜_2_ Female

**2. Nationality:**

⬜_1_ Kuwaiti ⬜_2_ Non-Kuwaiti

**3. Educational level:**

⬜_1_ Primary ⬜_2_  Secondary ⬜_3_ High school.

**4.** **Educational region:**

⬜_1_ Al-Farwaniyah ⬜_2_ Hawally

⬜_3_ Al-Asimah ⬜_4_ Al-Jahra

⬜_5_ Mubarak Al-Kabeer ⬜_6_ Al-Ahmadi

**5. Last semester’s overall grade in report:**

⬜_1_ A (90–100%) ⬜_2_ B (80–89%)

⬜_3_ C (70–79%) ⬜_4_ D (60–69%)

⬜_5_ F (less than 60%)

**6. Educational level of your father:**

⬜_1_Secondary school or lower ⬜_2_High school ⬜_3_ Diploma ⬜_4_ Bachelor ⬜_5_ Postgraduate

**7. Educational level of you mother:**

⬜_1_Secondary school or lower ⬜_2_High school ⬜_3_ Diploma ⬜_4_ Bachelor ⬜_5_ Postgraduate

**8. Family income (KD per month)**

⬜_1_ Less than 500 KD ⬜_2_ 500–1,000 KD ⬜_3_1,000–2,000 KD ⬜_4_ More than 2,000 KD

**Section 2: Student’s physical attributes**

**9. Weight:** ___________ **kg** **Height** ___________ **cm**

**10. Do you play any sport on a regular basis (three or more times per week)?**

⬜_1_ Yes

⬜_2_ No

**11.** **On a scale of 1–10, how physically active are you?** Please circle the number that match you (1 being not active at all and 10 being very active)

**1 2 3 4 5 6 7 8 9 10**

**Section 3: Student’s pattern of smart devise use**

**12. What type of smart device are you using?** (You can tick more than one option)

⬜_1_ Smartphones ⬜_2_ Tablets

**13. What is the average total hours of smart device use per day?**

⬜_1_ Less than 2 hours ⬜_2_ 2–4 hours ⬜_3_ More than 4 hours

**14. What is the average screen time spent on the smart device per session?**

⬜_1_ Less than 1 hour ⬜_2_ 1 hour- 2 hours ⬜_3_ More than 2 hours

**15. What is the most common time during the day for you to use smart devices**? (You can tick more than one option)

⬜_1_ Morning ⬜_2_ Evening

⬜_3_ Afternoon ⬜_4_ Bed time

**16. Please rank the following smart device activities from 1 to 7? from the activity you do the most (1) to the activity you do the least (7).**

⬜ Watching videos ⬜ Using educational applications

⬜ Using social media (e.g. what’s up, Snap) ⬜ Video calling

⬜ Playing games ⬜ Surfing the Internet

⬜ Doing school work

**Section 4: Health-related problems**

1. **Medical history**

**17. Have you been diagnosed by a physician with any of the following problems?** (You can tick more than one problem). If your answer is “None”, please go to the next question.

⬜ Seizures ⬜ Obesity

⬜ Headaches ⬜ Back injuries

⬜ Eye problems, if yes, please tick which of the following problems:

*Blurry vision* ⬜ *Eye dryness* ⬜ *Near-sightedness* ⬜

⬜ Others, please specify: ___________

⬜ None

**18. Do you have any family medical history (parents, brother or sisters) of the following?** If your answer is “None”, please go to the next question.

⬜ Seizures

⬜ Headache

⬜ Visual impairment

⬜ Others_,_ please specify: ___________

⬜ None

1. **Health problems**

**19. Have you experienced any of the following problem AFTER using smart devices?** (You can tick more than one problem).

⬜ Brain seizures ⬜ Blurred vision ⬜ Neck/shoulder pain

⬜ Near-sightedness ⬜ Transient vision loss ⬜ Lower-back pain

⬜ Eye flashes ⬜ Headache ⬜ Loss of concentration ⬜ Eye dryness ⬜ Sleep disturbance ⬜ Obesity

⬜ Eye Squint

⬜ **None**

**Thank you very much for your participation**
